# Dietary Inflammatory Potential Is Associated With Sarcopenia Among Chronic Kidney Disease Population

**DOI:** 10.3389/fnut.2022.856726

**Published:** 2022-05-11

**Authors:** Ying Huang, Mengru Zeng, Lei Zhang, Jingzheng Shi, Yuan Yang, Fuyou Liu, Lin Sun, Li Xiao

**Affiliations:** ^1^Department of Nephrology, The Second Xiangya Hospital of Central South University, Hunan Key Laboratory of Kidney Disease and Blood Purification, Changsha, China; ^2^Department of Epidemiology and Health Statistics, Xiangya School of Public Health, Central South University, Changsha, China; ^3^School of Public Health, Guilin Medical College, Guilin, China

**Keywords:** dietary inflammatory potential, dietary inflammation index, sarcopenia, chronic kidney disease, cross-section study, National Health and Nutrition Examination Survey

## Abstract

**Background:**

Sarcopenia, characterized by impaired muscle mass and function, is a common complication and the main reason for bad life quality and high mortality in chronic kidney disease (CKD). Limiting systemic inflammation is a potable intervention for sarcopenia. Dietary inflammatory potential can influence systemic inflammation. However, research about the association between dietary inflammatory potential and sarcopenia in CKD is limited.

**Aim:**

To investigate the association between dietary inflammatory potential and sarcopenia in the CKD population.

**Methods:**

We conducted a cross-section study based on the public database of the National Health and Nutrition Examination Survey (NHANES). In total, 2,569 adult CKD participants who had complete data for dietary inflammatory potential and sarcopenia were included. The dietary inflammatory potential was calculated by the dietary inflammation index (DII) score based on dietary recall interviews. We assessed sarcopenia *via* low skeletal muscle mass measured by dual-energy X-ray absorptiometry. Smooth curve fitting and a generalized linear mixed model were used to evaluate the relationship between DII and sarcopenia. Moreover, subgroup and sensitivity analyses were performed.

**Results:**

The overall prevalence of sarcopenia among patients with CKD is 19.11%. Smooth curve fitting results displayed that the DII score is near-linear positively associated with sarcopenia. Logistic regression confirmed sarcopenia is independently related to DII scores (odds ratio [OR], 1.17; 95% CI, 1.06–1.29). Subgroup analyses revealed relatively stronger associations between DII and sarcopenia among patients with CKD with other sarcopenia risk factors, such as hypoalbuminemia, low energy intake, low protein intake, and comorbidities.

**Conclusion:**

The dietary inflammatory potential is independently related to sarcopenia among patients with CKD. Anti-inflammatory diet patterns may be a protective intervention for CKD-associated sarcopenia.

## Introduction

Chronic kidney disease (CKD) has become one of the most common health issues, leading to increasing global healthcare burdens year by year (1). Sarcopenia, characterized by a loss of muscle mass and function, is a common complication of CKD. The prevalence of sarcopenia among patients with CKD is associated with CKD progression, complications, and therapies and related comorbidities. CKD patients with sarcopenia not only have a bad quality of life but also suffer higher morbidity and mortality (2–4).

In the past, the mechanisms of CKD-associated sarcopenia have mainly focused on the imbalance of protein synthesis and degradation, malnutrition, metabolic acidosis, abnormal insulin signal, and myostatin (4). Recently, it was found that low-grade local and systemic inflammation plays an important role in the development of sarcopenia (5). Besides, as we all know, systemic low-grade inflammation is a key feature of CKD. Sarcopenia and systemic inflammation commonly coexist, and the net skeletal muscle protein balance was found negatively associated with hs-CRP in patients with CKD (6). It was also demonstrated that uremic serum can activate TNF-α, inhibit pAkt, and finally decrease protein synthesis in C2C12 cells (7). Thus, limiting inflammation is a potential intervention for CKD-associated sarcopenia.

The CKD-associated inflammation is not only related to the uremic condition, but it is also influenced by many factors, such as exercise (2) and diet (8), both of which can be intervened in practice. Besides, adherence to a healthy diet plays a vital role in preventing sarcopenia. However, in the past, dietary interventions for sarcopenia mainly focused on a high-quality protein-rich diet and supplementary macro- and micronutrients (9, 10). The integral effect of dietary patterns and their role in adjusting systemic inflammation were neglected. It was discovered that dietary components and specific nutrients show an effect on inflammation and the dietary inflammatory index (DII) was developed to evaluate the total inflammatory potential of a diet (11). Recently, more and more studies reported that the DII score is highly associated with numerous inflammatory diseases, such as obesity, diabetes, cardiovascular disease, non-alcoholic fatty liver disease, periodontitis, and chronic kidney disease (8, 12–14). Many cross-sectional studies also found the DII score was positively associated with sarcopenia, such as older adult population (15–17). However, the association between dietary inflammatory potential and CKD-associated sarcopenia remains unclear. Thus, this study aimed to examine the relationship between DII score and sarcopenia among patients with CKD based on a representative sample from the public database, National Health and Nutrition Examination Survey (NHANES). We hypothesized that DII is positively associated with sarcopenia in the CKD population.

## Materials and Methods

### Study Population

The NHANES is a program conducted to evaluate people's health and nutritional status in the United States. It combines interviews and physical examinations to collect demographics, dietary, medical examination, laboratory, and questionnaire data (18). The National Center for Health Statistics Research Ethics Review Board approved data collection for NHANES, and written informed consent was obtained from participants.

**Figure 1:**
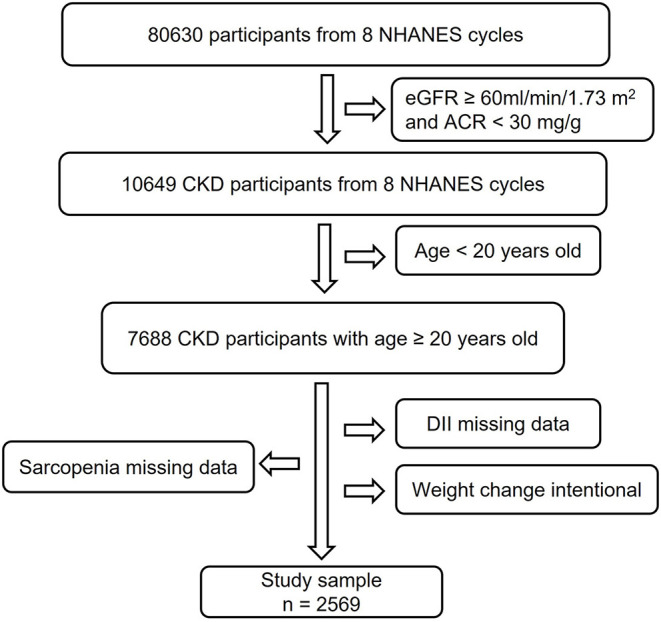
Study flowchart. In this study, 80,630 participants from 8 NHANES cycles were involved. Individuals with eGFR ≥60 ml/min/1.73 m^2^, ACR <30 mg/g, and age <20 years old did not meet the inclusion criteria. After excluding subjects with missing data for DII, sarcopenia, and intentional weight change history, 2,569 participants were finally used for analysis. CKD, Chronic Kidney Disease; NHANES, National Health and Nutrition Examination Survey; DII, Dietary Inflammatory Index; eGFR, estimate Glomerular Filtration Rate; ACR, urine Albumin to Creatinine Ratio.

In this study, we used data from 8 NHANES cycles: 1999–2000, 2001–2002, 2003–2004, 2005–2006, 2011–2012, 2013–2014, 2015–2016, and 2017–2018 (18). As shown in Figure 1, we collected 2,569 CKD participants with complete data for DII and sarcopenia, excluding individuals under 20 years old and with an intentional weight-changing history.

### Chronic Kidney Disease

We used the Modification of Diet in Renal Disease Study Equation to estimate glomerular filtration rate (eGFR) (19). CKD was defined as eGFR <60 ml/min per 1.73 m^2^ or urine albumin-to-creatinine ratio (*U*ACR) ≥30 mg/g (20).

### Exposure Variable

Dietary inflammatory index was the major exposure variable. The DII score estimates the impact of diet on inflammation based on the pro- and anti-inflammatory properties of 45 different food components, which were developed according to a systematic review of ~2,000 published research articles (11). Higher positive DII scores mean more pro-inflammatory diets, whereas more negative values correspond to more anti-inflammatory (11). We calculate DII based on dietary intake information by 24 h dietary recalls (24 HR) in this study. Moreover, 27 or 28 food components in 24 HR were used to calculate the DII scores: energy, carbohydrate; protein; total fat; dietary fiber; cholesterol; saturated, monounsaturated, and polyunsaturated fatty acids; ω-3 and ω-6 polyunsaturated fatty acids; vitamins A, B1, B2, B3 (niacin), B6, B12, C, D, and E; folic acid; alcohol; beta-carotene; caffeine; iron; magnesium; zinc; and selenium. Previous studies confirmed that DII scores based on only 27 or 28 food parameters did not influence the predictive ability (13, 21). Since we calculate DII scores according to participants' dietary recall information, we performed a consistency test to evaluate the accuracy of the measurement. As shown in Supplementary Table S1, there was no significant difference between DII scores from the first 24 HR and second 24HR (*p* = 0.2363).

### Outcome Variable

The outcome of interest was sarcopenia, which was assessed by the sum of four limbs' muscle mass (appendicular lean mass, ALM) (22). NHANES used dual-energy X-ray absorptiometry (DEXA) to measure ALM. Participants with a height > 192.5 cm, weight > 136.4 kg, and pregnant individuals were excluded since these participants could not perform the DEXA test (18). We defined sarcopenia by BMI-adjusted ALM (ALM_BMI_): men were judged as sarcopenia if ALM_BMI_ <0.789, and women < 0.512 (22).

### Potential Cofounders

Socio-demographic factors included age, gender, race, and income. We categorized race into 4 groups: Mexican American, non-Hispanic white, non-Hispanic black, and others. Income was evaluated by the poverty income ratio (PIR). PIR < 1 was considered as poor, 1–3 near-poor, and ≥3 as not poor (23). Behavioral variables were physical activity, cigarette smoking, and alcohol drinking. Physical activity was divided into inactive and active groups according to average physical activity time. Participants who met the American Physical Activity Council's chronic health conditions recommendation (75 min/week vigorous or 150 min/week moderate activity) were active (24). Individuals whose dietary alcohol intake was more than 0 g were alcohol consumers. Participants who self-reported smoking more than 100 cigarettes in life were identified as smokers.

Since protein-energy wasting and comorbidities could influence muscle atrophy, we considered low energy intake (energy intake <25 kal/kg/day), low protein intake (<0.6 g/kg/day), and hypoalbuminemia (<38 g/L) (25, 26). In total, 8 comorbidities were included in the analysis: diabetes, hypertension, overweight, central obesity, dyslipidemia, cardiovascular diseases, arthritis, and cancer (Supplementary Table S2) (14, 27). Biomarkers of inflammation included C-reactive protein (CRP), white blood cell (WBC) count, and neutrophil-lymphocyte ratio (NLR).

We collected data from 8 NHANES cycles, corresponding to a 20-year cross-section ranging from 1999 to 2018. Based on potential diet changes over 20 years, we also considered NHANES strata. Each NHANES strata contains two cycles: NHANES strata 1 (NHANES 1999–2002), NHANES strata 2 (NHANES 2003–2006), NHANES strata 3 (NHANES 2010–2014), and NHANES strata 4 (NHANES 2015–2018).

### Statistical Analyses

The characteristics of participants overall and among different DII groups (tertiles) are summarized in Table 1. Continuous variables with a normal distribution were presented as mean ± standard deviation (SD). Non-normal continuous variables were reported as median with an interquartile range. Categorical variables were presented as frequency and percentage. Comparisons among different groups were performed by one-way ANOVA tests for normally distributed variables, independent-samples Kruskal–Wallis tests for non-normal continuous variables, and chi-square tests for categorical variables.

**Table 1:** Characteristics of 2,569 patients with chronic kidney disease (CKD) aged ≥20 years from 8 NHANES cycles overall and by the tertile of DII.

	**Dietary Inflammatory Index**				
	**Overall**	**T1**	**T2**	**T3**	***P*** **-value**
	**(2569)**	**(856)**	**(856)**	**(857)**	
DII, min-max	−4.31-4.86	−4.31-0.68	0.68-2.41	2.41-4.86	
Age, year, mean (SD)	55.6 ± 18.1	54.7 ± 18.0	56.0 ± 17.8	56.1 ± 18.5	0.205
Gender-male, *n* (%)	1159 (45.11%)	476 (55.61%)	400 (46.73%)	283 (33.02%)	<0.001
**Race, *n* (%)**					<0.001
Mexican American	475 (18.49%)	181 (21.14%)	147 (17.17%)	147 (17.15%)	
Non-Hispanic White	1,209 (47.06%)	424 (49.53%)	398 (46.50%)	387 (45.16%)	
Non-Hispanic Black	508 (19.77%)	112 (13.08%)	182 (21.26%)	214 (24.97%)	
other	377 (14.67%)	139 (16.24%)	129 (15.07%)	109 (12.72%)	
Income, *n* (%)					<0.001
poor	515 (21.88%)	133 (17.10%)	167 (21.30%)	215 (27.15%)	
near poor	1,074 (45.62%)	350 (44.99%)	373 (47.58%)	351 (44.32%)	
not poor	765 (32.50%)	295 (37.92%)	244 (31.12%)	226 (28.54%)	
Physical activities-active, *n* (%)	1,283 (54.62%)	476 (60.10%)	415 (53.48%)	392 (50.19%)	<0.001
Smoking, *n* (%)	1,258 (49.06%)	412 (48.30%)	419 (48.95%)	427 (49.94%)	0.792
Drinking, *n* (%)	578 (22.50%)	238 (27.80%)	205 (23.95%)	135 (15.75%)	<0.001
Diabetes, *n* (%)	692 (26.94%)	224 (26.17%)	239 (27.92%)	229 (26.72%)	0.705
Hypertension, *n* (%)	1,508 (62.70%)	449 (56.12%)	529 (66.37%)	530 (65.59%)	<0.001
Overweight, (*n*%)	1,779 (69.25%)	573 (66.94%)	609 (71.14%)	597 (69.66%)	0.161
Central obesity, *n* (%)	1,972 (77.52%)	643 (75.20%)	667 (79.03%)	662 (78.34%)	0.131
Dyslipidaemia, *n* (%)	1,672 (65.83%)	552 (65.09%)	581 (68.68%)	539 (63.71%)	0.085
**Cancer, *n* (%)**	269 (10.48%)	86 (10.06%)	96 (11.21%)	87 (10.18%)	0.691
Arthrities, *n* (%)	805 (31.46%)	238 (27.93%)	270 (31.73%)	297 (34.70%)	0.011
Heart Diseases, *n* (%)	469 (18.26%)	138 (16.12%)	162 (18.93%)	169 (19.72%)	0.129
Hypoalbuminemia, *n* (%)	208 (8.43%)	62 (7.51%)	55 (6.72%)	91 (11.08%)	0.003
Low energy intak, *n* (%)	1,460 (56.83%)	254 (29.67%)	518 (60.51%)	688 (80.28%)	<0.001
Low protein intak, *n* (%)	610 (23.74%)	43 (5.02%)	157 (18.34%)	410 (47.84%)	<0.001
**eGFR**					0.065
G1	851 (34.51%)	307 (37.17%)	266 (32.48%)	278 (33.86%)	
G2	626 (25.39%)	219 (26.51%)	213 (26.01%)	194 (23.63%)	
G3–G4	989 (40.11%)	300 (36.32%)	340 (41.51%)	349 (42.51%)	
**ACR**					0.729
A1	688 (27.01%)	222 (26.12%)	236 (27.80%)	230 (27.12%)	
A2	1,564 (61.41%)	534 (62.82%)	507 (59.72%)	523 (61.67%)	
A3	295 (11.58%)	94 (11.06%)	106 (12.49%)	95 (11.20%)	
CRP, mg/dL, median(Q1, Q3)	0.26 (0.11-0.57)	0.20 (0.09–0.46)	0.26 (0.12–0.55)	0.32 (0.15–0.69)	<0.001
WBC, 1,000/μL, mean(SD)	7.43 ± 2.18	7.51 ± 2.20	7.43 ± 2.18	7.36 ± 2.17	0.397
NLR, mean(SD)	2.43 ± 1.42	2.50 ± 1.39	2.38 ± 1.34	2.42 ± 1.53	0.181
**NHANES strata**					0.035
1999–2002	913 (35.54%)	303 (35.40%)	286 (33.41%)	324 (37.81%)	
2003–2006	806 (31.37%)	243 (28.39%)	295 (34.46%)	268 (31.27%)	
2010–2014	470 (18.30%)	168 (19.63%)	162 (18.93%)	140 (16.34%)	
2016–2018	380 (14.79%)	142 (16.59%)	113 (13.20%)	125 (14.59%)	
Sarcopenia, *n* (%)	491 (19.11%)	152 (17.76%)	152 (17.76%)	187 (21.82%)	0.047

*CKD, Chronic Kidney Disease; NHANES, National Health and Nutrition Examination Survey; DII, Dietary Inflammatory Index; T1, Tertile 1; T2, Tertile 2; T3, Tertile 3; SD, standard deviation; eGFR, estimate Glomerular Filtration Rate; ACR, urine Albumin to Creatinine Ratio; CRP, C Reactive Protein; WBC, White Blood Cell; NLR, Neutrophil-Lymphocyte Ratio. Missing values for total study: income (n = 215; 8.4%), physical activity (n = 220; 8.6%), Smoking (n = 5; 0.2%), hypertension (n = 164; 6.4%), central obesity (n = 25; 1.0%), dyslipidemia (n = 29; 1.1%), cancer (n = 3; 0.1%), arthritis (n = 10; 0.4%), hypoalbuminemia (n = 103; 4.0%), eGFR (n = 103; 4.0%), ACR (n = 22; 0.9%), CRP (n = 549; 21.4%), WBC (n = 62; 2.4%) and NLR (n = 79; 3.1%)*.

To evaluate the association between dietary inflammatory potential and sarcopenia, we first conducted smooth curve fitting (penalized spline method) to address the non-linearity of DII and sarcopenia. Then, a generalized linear mixed model (GLMM) was applied to determine the independent association between DII and sarcopenia. Model 1 had no covariate adjusted; model 2 had age, gender, and race adjusted; model 3 had adjusted for age, gender, race, income, physical activity, smoking, alcohol drinking, diabetes, hypertension, overweight, central obesity, dyslipidemia, cancer, arthritis, heart disease, eGFR, ACR, hypoalbuminemia, low energy intake, low protein intake, CRP, WBC, NLR, and NHANES strata. In addition, we performed subgroup analyses. All continuous covariables were converted into tertiles categorical variables and the interaction effect was assessed *via* the likelihood ratio test. A sensitivity analysis was conducted by converting DII into a tertile categorical variable.

All analyses were conducted with Empower (R) (28) and RStudio (29).

## Results

### Characteristics of the Study Sample

As described in Figure 1, 2,569 participants from 8 NHANES cycles were included in this study. Table 1 summarized the characteristics of participants overall and among DII tertiles groups. On average, participants were 55.6 years old. About 45.11% were men. The overall prevalence of sarcopenia among patients with CKD was 19.11%. The overall DII score ranged from −4.31 to 4.86. We equally divided the subjects into 3 groups based on their DII score: the first tertile group (T1, *n* = 856; DII = −4.31 to 0.68), the second tertile group (T2, *n* = 856; DII = 0.68–2.41), and the third one (T3, *n* = 857; DII = 2.41–4.86). Subjects in the T1 group consumed a more anti-inflammatory diet, whereas participants in the T3 group intake more pro-inflammatory diet. Compared with the T1 group, subjects in the T3 group were more likely to be female, Non-Hispanic black, poor, inactive, and non-alcohol consumers. Additionally, they had a higher incidence of hypertension, arthritis, low energy intake, low protein intake, and hypoalbuminemia than those in the T1 group (*p* < 0.05). Besides, the level of CRP in the T3 group was significantly higher than in the T1 group, which confirmed that consuming more pro-inflammatory food is positively associated with systematic inflammation.

### Dietary Inflammation and Sarcopenia

**Figure 2:**
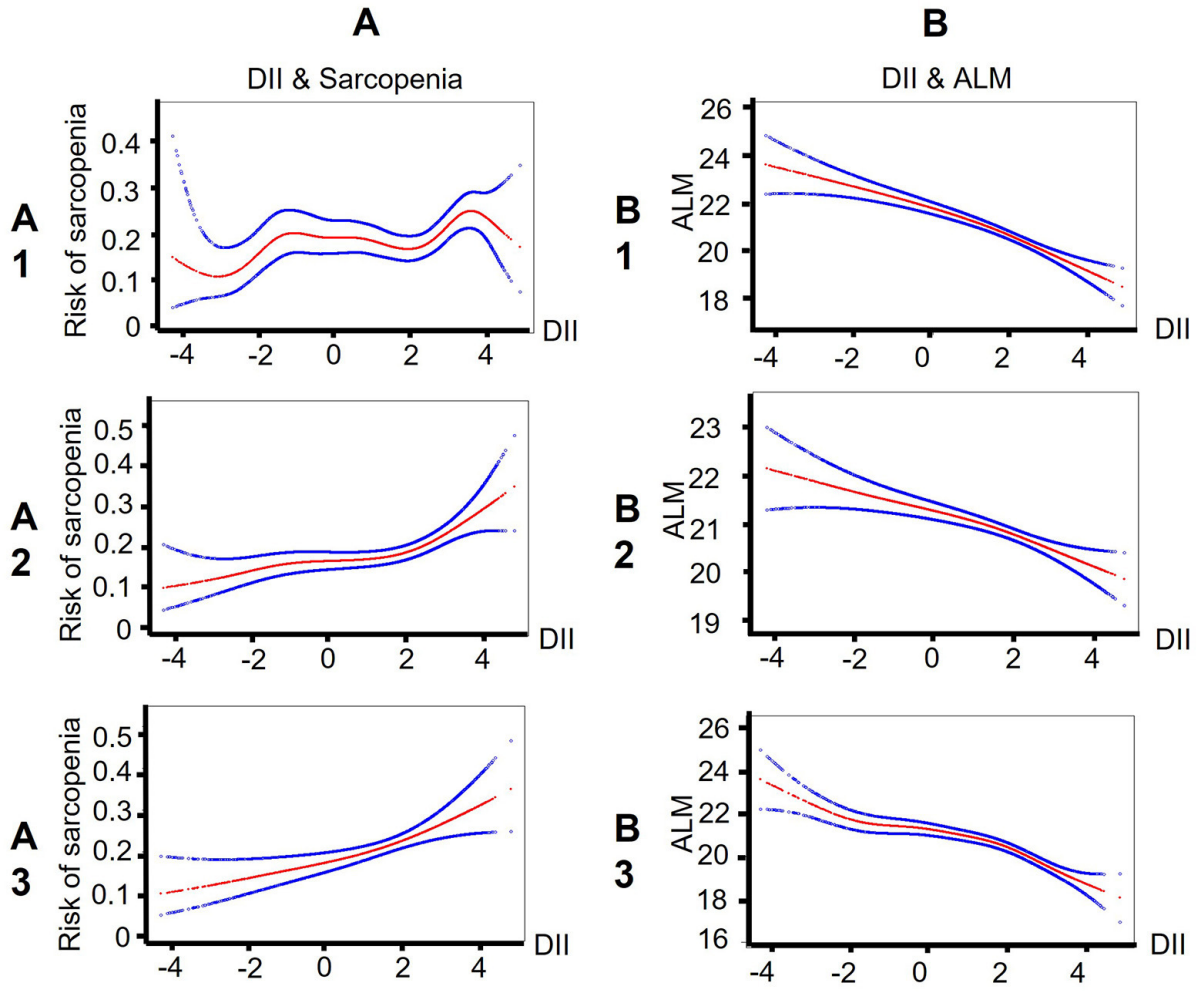
Smooth curve fitting results indicate that the DII score is near-linear associated with sarcopenia/ALM among patients with CKD. **(A)** Smooth curve fitting results between DII and sarcopenia. Risk of sarcopenia (red) with 95% CIs (blue) determined using the generalized additive model. **(B)** Smooth curve fitting results between DII and ALM. ALM (red) with 95% *CI*s (blue) determined using the generalized additive model. A1/B1: no adjustment; A2/B2: adjusted for age, gender, and race; A3/B3: adjusted for age, gender, race, income, physical activity, smoking, alcohol drinking, diabetes, hypertension, overweight, central obesity, dyslipidemia, cancer, arthritis, heart disease, eGFR, ACR, hypoalbuminemia, low energy intake, low protein intake, CRP, WBC, NLR, *and NHANES strata*. DII, Dietary Inflammatory index; ALM, appendicular lean mass; CKD, Chronic Kidney Disease; eGFR, estimate Glomerular Filtration Rate; ACR, urine Albumin to Creatinine Ratio; CRP, C Reactive Protein; WBC, White Blood Cell; NLR, Neutrophil-Lymphocyte Ratio.

The smooth curve fitting results showed the DII score is near-linear positively associated with sarcopenia after adjusting for cofounders (Figure 2A). Then, the generalized linear mixed model analysis further confirmed that sarcopenia is positively related with DII scores (Table 2). The odds ratio (*OR*) is 1.07 (95% *CI*, 1.02–1.13), 1.15 (95% *CI*, 1.08–1.22), and 1.17 (95% CI, 1.06–1.29), respectively, in the model 1 (no adjustment), model 2 (adjusted for age, gender, and race), and model 3 (adjusted for all cofounders). To ensure the validity of the results, we also performed a sensitivity analysis by converting DII to a categorical variable (tertiles). The new adjusted *OR* values were 1.27 (95% *CI*, 0.88, 1.84) for T2, and 1.98 (95% CI, 1.32, 2.98) for T3 (a reference to T1), and the p for trend was 0.001.

**Table 2:** Associations between the DII and sarcopenia among patients with CKD aged ≥20 years old.

	**OR (95% CI), *P*-value**		
	**Model 1**	**Model 2**	**Model 3**
DII continuous	1.07 (1.02, 1.13) 0.012	1.15 (1.08, 1.22) <0.0001	1.17 (1.06, 1.29) 0.001
**DII Tertile^@^**			
T1	1	1	1
T2	1.00 (0.78, 1.28) 1.000	1.15 (0.88, 1.50) 0.302	1.27 (0.88, 1.84) 0.200
T3	1.29 (1.02, 1.64) 0.035	1.74 (1.33, 2.26) <0.0001	1.98 (1.32, 2.98) 0.001

*Model 1: no adjustment*.

*Model 2: adjusted for age, gender, and race*.

*Model 3: adjusted for age, gender, race, income, physical activity, smoking, alcohol drinking, diabetes, hypertension, overweight, central obesity, dyslipidemia, cancer, arthritis, heart disease, eGFR, ACR, hypoalbuminemia, low energy intake, low protein intake, CRP, WBC, NLR, and NHANES strata*.

@ *p for trend in model 1 is 0.033; in model 2 is <0.001; in model 3 is 0.001*.

*CKD, Chronic Kidney Disease; DII, Dietary Inflammatory Index; T1, Tertile 1; T2, Tertile 2; T3, Tertile 3; OR, Odds Ratio; CI, Confidence Interval; eGFR, estimate Glomerular Filtration Rate; ACR, urine Albumin to Creatinine Ratio; CRP, C Reactive Protein; WBC, White Blood Cell; NLR, Neutrophil-Lymphocyte Ratio; NHANES, National Health and Nutrition Examination Survey*.

In addition, we also assessed the relationship between DII and ALM. The smooth curve fitting figure displayed that the DII score has a linear negative relationship with ALM (Figure 2B). The GLMM analysis also showed that DII is independently related with ALM (Table 3). The OR value was −0.50 (95% CI, −0.59, −0.40) after adjusting for all cofounders.

**Table 3:** Associations between the DII and ALM among patients with CKD aged ≥20 years old.

	**β (95% CI), *P*-value**		
	**Model 1**	**Model 2**	**Model 3**
**DII**	−0.59 (−0.71, −0.46) <0.0001	−0.24 (−0.33, −0.15) <0.0001	−0.50 (−0.59, −0.40) <0.0001

*Model 1: no adjustment*.

*Model 2: adjusted for age, gender and race*.

*Model 3: adjusted for age, gender, race, income, physical activity, smoking, alcohol drinking, diabetes, hypertension, overweight, central obesity, dyslipidemia, cancer, arthritis, heart disease, eGFR, ACR, hypoalbuminemia, low energy intake, low protein intake, CRP, WBC, NLR, and NHANES strata*.

*CKD, Chronic Kidney Disease; DII, Dietary Inflammatory Index; ALM, appendicular lean mass; CI, Confidence Interval; eGFR, estimate Glomerular Filtration Rate; ACR, urine Albumin to Creatinine Ratio; CRP, C Reactive Protein; WBC, White Blood Cell; NLR, Neutrophil-Lymphocyte Ratio; NHANES, National Health and Nutrition Examination Survey*.

### Subgroup Analyses

Subgroup analyses results are summarized in Table 4. Relatively stronger associations between DII and sarcopenia were observed among women, older adults, active subjects, smokers, non-alcohol drinkers, and patients with hypoalbuminemia, low energy intake, low protein intake, diabetes, overweight, central obesity, non-dyslipidemia, non-cancer, and non-heart disease. Patients with CKD in *G3-5* and A3 stages were also more likely to be sarcopenia if they consumed more pro-inflammatory diets. However, only low energy intake, low protein intake, and dyslipidemia have significant interactions with DII (*p* for interaction < 0.05). Besides, the association between DII and sarcopenia also remains consistent and stronger in subjects with high levels of CRP and NLR. Significant interactions between CRP and DII were also observed, with *p* for interaction < 0.05.

**Table 4:** Subgroup analysis.

**Exposure**	**OR, 95%CI**	***p*** **-value**	***p*** **for interaction**
**Gender**			0.715
man	1.11 (0.98 1.27)	0.114	
Women	1.28 (1.09–1.50)	0.002	
**Age**			0.953
Tertile 1	1.13 (0.85–1.54)	0.403	
Tertile 2	1.12 (0.93–1.37)	0.234	
Tertile 3	1.19 (1.05–1.36)	0.008	
**Race**			0.757
Mexican American	1.12 (0.93–1.35)	0.252	
Non-Hispanic White	1.19 (1.04–1.38)	0.015	
Non-Hispanic Black	1.44 (0.79–3.12)	0.274	
Other	1.25 (0.89–1.79)	0.204	
**Income**			0.795
Poor	1.17 (0.91–1.52)	0.220	
Near poor	1.18 (1.02–1.36)	0.026	
Not poor	1.22 (1.02–1.48)	0.034	
**Physical Activity**			0.434
Active	1.24 (1.07–1.45)	0.006	
Inactive	1.12 (0.98–1.28)	0.104	
**Alcohol Drinking**			0.683
Yes	1.10 (0.86–1.41)	0.453	
No	1.18 (1.06–1.32)	0.003	
**Smoking**			0.437
Yes	1.24 (1.09–1.43)	0.002	
No	1.12 (0.96–1.30)	0.147	
**Diabetes**			0.497
Yes	1.24 (1.04–1.49)	0.021	
No	1.15 (1.02–1.30)	0.025	
**Hypertension**			0.811
Yes	1.16 (1.03–1.30)	0.012	
No	1.28 (1.05–1.58)	0.016	
**Overweight**			0.448
Yes	1.24 (1.11–1.39)	<0.001	
No	0.95 (0.74–1.21)	0.651	
**Central Obesity**			0.490
Yes	1.20 (1.08–1.33)	0.001	
No	0.85 (0.55–1.31)	0.459	
**Dyslipidemia**			**0.019**
Yes	1.12 (1.00–1.25)	0.057	
No	1.42 (1.16–1.76)	0.001	
**Cancer**			0.823
Yes	1.07 (0.80–1.47)	0.637	
No	1.18 (1.06–1.31)	0.003	
**Arthritis**			0.730
Yes	1.21 (1.03–1.43)	0.025	
No	1.14 (1.01–1.30)	0.036	
**Heart Disease**			0.124
Yes	1.11 (0.90–1.37)	0.332	
No	1.24 (1.11–1.40)	<0.001	
**Hypoalbuminemia**			0.390
Yes	1.66 (1.07–2.80)	0.035	
No	1.16 (1.05–1.29)	0.004	
**Low energy intake**			**0.012**
Yes	1.31 (1.14–1.52)	<0.001	
No	1.04 (0.90–1.21)	0.563	
**Low protein intake**			**0.003**
Yes	2.06 (1.49–2.91)	<0.001	
No	1.08 (0.97–1.20)	0.141	
**eGFR**			0.381
G1	1.20 (1.00–1.44)	0.055	
G2	1.15 (0.94–1.43)	0.178	
G3–G5	1.22 (1.05–1.43)	0.010	
**ACR**			0.557
A1	1.10 (0.92–1.32)	0.284	
A2	1.17 (1.03–1.33)	0.016	
A3	1.85 (1.21–2.98)	0.007	
**CRP**			**0.027**
Tertile 1	1.11 (0.92–1.36)	0.288	
Tertile 2	1.09 (0.92–1.30)	0.308	
Tertile 3	1.43 (1.21–1.72)	<0.001	
**WBC**			**0.020**
Tertile 1	1.37 (1.13–1.67)	0.002	
Tertile 2	1.16 (0.97–1.39)	0.119	
Tertile 3	1.11 (0.94–1.32)	0.222	
**NLR**			0.629
Tertile 1	1.11 (0.92–1.36)	0.282	
Tertile 2	1.16 (0.97–1.39)	0.119	
Tertile 3	1.20 (1.02–1.42)	0.029	

*Age (years old), Tertile 1: 20–47, Tertile 2: 48–64; Tertile 3: 65–85; CRP (mg/dl), Tertile 1: 0.01–0.14, Tertile 2: 0.15–0.42; Tertile 3: 0.43–29.6; WBC count (1,000/ul), Tertile 1: 2.3-6.3, Tertile 2: 6.4–7.9; Tertile 3: 8.0–23.0; NLR, Tertile 1: 0.258–1.765, Tertile 2: 1.767–2.580; Tertile 3: 2.582–17.72*.

*The subgroup analysis was adjusted for all presented covariates except effect modifier. DII, Dietary Inflammatory Index; OR, Odds Ratio; CI, Confidence Interval; eGFR, estimate Glomerular Filtration Rate; ACR, urine Albumin to Creatinine Ratio; CRP, C Reactive Protein; WBC, White Blood Cell; NLR, Neutrophil-Lymphocyte Ratio. The bold values indicates p < 0.05*.

To figure out the influence of NHANES strata on the association effect between DII and sarcopenia, we also performed a subgroup analysis for NHANES strata. As shown in Supplementary Table S3, significant associations were only observed in NHANES strata 2003–2006 (DII continuous) and NHANES strata1999–2002 (DII tertile). However, p for heterogeneity was over 0.05, meaning there was no significant difference between 4 NHANES strata for relationships between DII and sarcopenia.

## Discussion

This study found that patients with CKD who consumed more pro-inflammatory diets were more likely to have sarcopenia. The smooth curve fitting results revealed that sarcopenia is near-linear related with DII. The generalized linear mixed model displayed that sarcopenia is independently associated with DII. Sensitivity analysis further confirmed the relationship between sarcopenia and DII. Besides, these results were consistent and relatively stronger among patients with CKD with other sarcopenia risk factors, such as old age, hypoalbuminemia, low energy intake, low protein intake, diabetes, overweight, and central obesity.

Sarcopenia is one of the most common complications of CKD. Previous studies found that the prevalence of sarcopenia in the CKD population ranged from 4 to 42% (30). Our study found the prevalence of sarcopenia to be 19.11%, consistent with previous reports. Although the mechanism of sarcopenia is complicated, more and more studies confirmed that inflammation participates in the occurrence and progression of sarcopenia (31, 32). It was reported inflammatory biomarkers, such as interleukin-6 and hsCRP, are significantly correlated with dialysis-associated sarcopenia (6, 33). These proinflammatory cytokines can promote protein degradation and prevent albumin synthesis, leading to protein-energy wasting (34). Besides, intervention for lowering systematic inflammation, such as exercise, may contribute to substantial improvements in muscle size among patients with CKD (2). Our study confirmed that systematic inflammation could be regulated by diets, as our results showed an anti-inflammatory diet is related with low levels of serum CRP. In addition, the independent relationship between sarcopenia and dietary inflammatory potential is significant among CKD patients with high levels of CRP and NLR, whereas the same phenomenon was not shown in subjects with low levels of CRP and NLR. It may reveal that a pro-inflammatory diet promotes muscle mass loss *via* deteriorating systematic inflammation, and protective dietary patterns should be a modifiable protective factor for CKD-associated sarcopenia. In addition, we found patients with CKD with other sarcopenia risk factors, such as protein-energy wasting and comorbidities, were easier influenced by dietary inflammatory potential, indicating we should strengthen dietary interventions for these patients to prevent sarcopenia. However, the relationship between DII and sarcopenia among CKD patients with cancer and heart disease is relatively weaker. As we all know, cancer and heart disease are severe cachexia diseases. The prevalence of sarcopenia in CKD patients with cancer (25.3%) and heart disease (30.1%) is significantly increased. The effect of diet may be diminished among these patients.

There are some limitations to this study. First, cross-section research only allows us to investigate the association between DII and sarcopenia instead of a casual relationship. Further prospective studies or even clinical trials are needed to confirm this. Second, sarcopenia is the decline in skeletal muscle mass with functional deterioration. In this study, we only evaluated sarcopenia *via* low muscle mass and did not consider muscle function due to lack of related information in NHANES datasets. Thus, studies involving the association between muscle function and DII also need to be conducted in the future. Third, CKD was judged by a single UACR and creatinine measurement. Participants might be falsely classified as CKD. Fourth, the diet information was from one-time recall surveys, which may not represent a usual diet. To minimize the influence of one-time recall bias and ensure the accuracy of diet information, we performed a consistency test between the first 24 HR and second 24 HR. The results showed the DII score from the first 24 HR is consistent with the second 24 HR. Despite the limitations, there are some strengths in this study. Most notably, this is the first time to confirm the relationship between DII and sarcopenia among patients with CKD based on the public NHANES database. The large and non-institutionalized samples make our results more convincing and applicable. Furthermore, we performed multivariate regression and subgroup analysis to exclude the influence of social demographics and some sarcopenia risk factors. Besides, subgroup analysis also revealed CKD patients with other sarcopenia high-risk achieve a better prevention on sarcopenia.

## Conclusion

This study determined dietary inflammatory potential is independently related with sarcopenia among the CKD population. Adherence to pro-inflammatory diet patterns should be a risk factor for CKD-associated sarcopenia, especially for patients with CKD who already have risk factors for low muscle mass. We should advocate healthy and anti-inflammatory diet patterns for patients with CKD to achieve better prevent sarcopenia.

## Data Availability Statement

Publicly available datasets were analyzed in this study. This data can be found here: https://www.cdc.gov/nchs/nhanes.

## Ethics Statement

The studies involving human participants were reviewed and approved by The National Center for Health Statistics Research Ethics Review Board. The patients/participants provided their written informed consent to participate in this study.

## Author Contributions

YH and LX designed the study. YH and LZ collected and organized the original data. YH and MZ analyzed the data. MZ, JS, and YY assisted with statistical analysis. YH, MZ, LZ, LS, FL, and LX assisted in the interpretation of the results and writing the manuscript. All authors contributed to the article and approved the submitted manuscript.

## Funding

This work was supported by the National Natural Science Foundation of China (grant number 82170744).

## Conflict of Interest

The authors declare that the research was conducted in the absence of any commercial or financial relationships that could be construed as a potential conflict of interest.

## Publisher's Note

All claims expressed in this article are solely those of the authors and do not necessarily represent those of their affiliated organizations, or those of the publisher, the editors and the reviewers. Any product that may be evaluated in this article, or claim that may be made by its manufacturer, is not guaranteed or endorsed by the publisher.
