# Supplementary material for: Dietary Inflammatory Potential Is Associated With Sarcopenia Among Chronic Kidney Disease Population
Source: Front Nutr. 2022 May 11;9:856726. doi: 10.3389/fnut.2022.856726 (PMC9131018; doi:10.3389/fnut.2022.856726)
Supplement: Supplementary file 1 [file Table_1.DOCX]

|  | **Table S1**. one sample t-test result | |
| --- | --- | --- |
|  | **Mean, 95%CI** | **p value** |
| **ΔDII** | 0.009 (-0.006, 0.025) | 0.2363 |

ΔDII is equal to DII from first 24HR minus DII from second 24HR

Abbreviations: DII, dietary inflammatory index; 24HR, 24 hour dietary recall interview

| **Table S2** Categorizations and definitions of comorbidities | | |
| --- | --- | --- |
|  | **Measurement, Units** | **Categorization Definitions** |
| **Diabetes** | Glycated hemoglobin level, %; Fasting blood glucose, mmol/L; Two Hour Glucose (OGTT), mmol/L) | Normal is no self-reported physician's diagnosis and no application of insulin or drugs to lower blood glucose and glycated hemoglobin level < 6.5% and Fasting blood glucose <7mmol/L; Two Hour Glucose <11.1 mmol/L; Diabetes is self-reported physician's diagnosis or application of insulin or drugs to lower blood glucose or glycated hemoglobin level ≥ 6.5% or Fasting blood glucose ≥ 7mmol/L; Two Hour Glucose ≥ 11.1 mmol/L |
|  | Self-reported physician's diagnosis or application of insulin or drugs to lower blood glucose |  |
| **Hypertension** | Systolic blood pressure (SBP), mm Hg; and diastolic blood pressure (DBP), mm Hg; | Normal is no self-reported physician's diagnosis, no application of hypertensive drugs and SBP < 140 mm Hg and DBP < 90 mm Hg; Hypertension is self-reported physician's diagnosis or application of hypertensive drugs or SBP ≥ 140 mm Hg or DBP ≥ 90 mm Hg. |
|  | Self-reported physician's diagnosis or anti-hypertensive drugs |  |
| **Overweight** | Body mass index (BMI), kg/m2 | Normal is BMI < 25 kg/m^2^; Overweight is BMI≥ 25 kg/m^2^ [1] |
| **Central obesity** | Waist circumstance, cm | Normal is waist circumstance < 85cm(women) /90cm (men); central obesity is waist circumstance ≥ 85cm(women)/90cm (men)[1] |
| **Dyslipidemia** | HDL cholesterol, mmol/L; triacylglycerol, mmol/L; LDL cholesterol, mmol/L | Normal is HDL cholesterol level < 1.0 mmol/L(men)/1.2mmol/L(women), triacylglycerol <1.7mmol/L and LDL cholesterol<3.0 mmolL; Dyslipidemia is self-reported physician's diagnosis or application of anti-dyslipidemia drugs or HDL cholesterol level ≥1.0 mmol/L(men)/1.2mmol/L(women) or triacylglycerol ≥ 1.7mmol/L or LDL cholesterol ≥ 3.0 mmolL. [1] |
|  | Self-reported physician's diagnosis or anti-dyslipidemia drugs |  |
| **Cancer** | Self-reported physician's diagnosis | Normal is no self-reported physician's diagnosis; cancer is self-reported physician's diagnosis.[2] |
| **Arthritis** | Self-reported physician's diagnosis | Normal is no self-reported physician's diagnosis; Arthritis is self-reported physician's diagnosis.[2] |
| **Heart disease** | Self-reported physician's diagnosis | Normal is no self-reported physician's diagnosis; heart disease is self-reported physician's diagnosis of congestive heart failure or coronary heart disease or angina pectoris or heart attack or stroke.[2] |

| **Table S3**. Subgroup analysis in different NHANES strata | | | | | |
| --- | --- | --- | --- | --- | --- |
|  |  | OR (95% CI) | p value | p for interaction | p for heterogeneity |
| **DII continuous** |  |  |  |  |  |
| NHANES strata |  |  |  | 0.860 | 0.352 |
| 1999-2002 (n=913) |  | 1.14(0.98, 1.33) | 0.087 |  |  |
| 2003-2006 (n=806) |  | 1.22(1.04, 1.44) | 0.014 |  |  |
| 2010-2014 (n=470) |  | 0.93(0.68, 1.27) | 0.654 |  |  |
| 2016-2018 (n=380) |  | 0.90(0.61, 1.32) | 0.585 |  |  |
| **DII Tertile^@^** |  |  |  |  |  |
| NHANES strata |  |  |  | 0.754 | 0.324 |
| 1999-2002 (n=913) | T1 | 1 |  |  |  |
|  | T2 | 1.31(0.72, 2.37) | 0.374 |  |  |
|  | T3 | 1.91(1.01, 3.63) | 0.048 |  |  |
| 2003-2006 (n=806) | T1 | 1 |  |  |  |
|  | T2 | 1.37(0.79, 2.42) | 0.265 |  |  |
|  | T3 | 1.85(0.99, 3.47) | 0.054 |  |  |
| 2010-2014 (n=470) | T1 | 1 |  |  |  |
|  | T2 | 1.15(0.31, 4.18) | 0.828 |  |  |
|  | T3 | 1.15(0.28, 4.59) | 0.846 |  |  |
| 2016-2018 (n=380) | T1 | 1 |  |  |  |
|  | T2 | 0.40(0.06 2.28) | 0.312 |  |  |
|  | T3 | 2.92(0.45, 19.63) | 0.259 |  |  |

**^@^**: DII Tertile 1: -4.3140~0.6766; DII Tertile 2: 0.6776~2.4077, DII Tertile 3: 2.4090~4.8630

Abbreviations: NHANES, National Health and Nutrition Examination Survey; DII, Dietary Inflammatory Index; T1, Tertile 1; T2, Tertile 2; T3, Tertile 3; OR, Odds Ratio; CI, Confidence Interval

[1] E. Han, Y.H. Lee, Y.D. Kim, B.K. Kim, J.Y. Park, D.Y. Kim, S.H. Ahn, B.W. Lee, E.S. Kang, B.S. Cha, K.H. Han, H.S. Nam, J.H. Heo, and S.U. Kim, Nonalcoholic Fatty Liver Disease and Sarcopenia Are Independently Associated With Cardiovascular Risk. Am J Gastroenterol 115 (2020) 584-595.

[2] A. Li, Y. Chen, A.A. Schuller, L.W.M. van der Sluis, and G.E. Tjakkes, Dietary inflammatory potential is associated with poor periodontal health: A population-based study. J Clin Periodontol 48 (2021) 907-918.
